# Brain connectivity signatures of cognitive impairment in temporal lobe epilepsy identified by robotic assessment

**DOI:** 10.1016/j.ynirp.2026.100330

**Published:** 2026-02-25

**Authors:** Karla Batista Garcia-Ramo, Spencer Finn, Theodore S. Aliyianis, Adam Falah, Brooke C. Beattie, Donald Brien, Garima Shukla, Lysa Boissé-Lomax, Stephen H. Scott, Jason P. Gallivan, Gavin P. Winston

**Affiliations:** a Centre for Neuroscience Studies, Queen's University, Kingston, ON, Canada; b Department of Medicine, Queen's University, Kingston, ON, Canada; c Department of Biomedical and Molecular Sciences, Queen's University, Kingston, ON, Canada; d Department of Psychology, Queen's University, Kingston, ON, Canada

**Keywords:** Epilepsy, Brain networks, Cognition, Robot Kinarm

## Abstract

**Background:**

Subjects with temporal lobe epilepsy (TLE) often experience cognitive impairment in different domains. Currently, the mechanisms underlying neuropsychological dysfunction in TLE remain poorly understood. The main objective is to characterize the multivariate relationship between brain connectivity patterns and cognitive impairment detected by robotic testing in subjects with TLE.

**Methods:**

Kinarm robotic technology was used to evaluate motor, cognitive, and sensory domains of healthy controls and individuals with TLE. Structural connectivity (SC) and functional connectivity (FC) were obtained from multi-shell diffusion MRI and resting-state fMRI, respectively. After principal component analysis for dimension reduction of connectivity features, sparse canonical correlation analyses were used to identify the patterns of multivariate association between brain connectivity and cognitive dysfunctions.

**Results:**

Patients with TLE demonstrated worse performance mainly in the domains of memory, executive function and attention, and to a lesser extent in the perceptual-motor domain. We found that memory and executive function alterations were associated with an intra-hemispheric SC pattern between somatomotor network and default, limbic and frontoparietal networks. We also found that an intra-hemispheric SC pattern of the posterior parietal cortex was related to perceptual-motor and attention skills with FC between this region and the precentral ventral region of DAN and frontal operculum insula of VAN also associated to impairment in these domains.

**Conclusions:**

This study identifies multivariate patterns of structural and functional connectivity that correlate with domain-specific cognitive impairment, as measured by robotic screening, in individuals with TLE. These findings support the conceptualization of TLE as a network disorder, contextualizing multidomain cognitive deficits within a network-level framework rather than interrogating specific functional circuits. This may in the future permit more personalized treatments or prediction of cognitive changes in response to planned treatment changes.

## Highlights

• Multivariate associations between brain connectivity and cognitive performance with robotics were studied in TLE.
• Memory/executive function associated with SC between somatomotor network and default, limbic and frontoparietal networks.
• Perceptual-motor/attention associated with FC between dorsal attention and somatomotor/ventral attention network.
• Kinarm robot shows promise as a cognitive screening tool for patients with TLE.

## 1 Introduction

Epilepsy is a disorder of cognition and behavior in addition to seizures (Fisher et al., 2005). The conceptualization of epilepsy as a network disorder encompasses changes in underlying brain neural networks that extend beyond any specific identifiable lesion in focal epilepsy (Kramer and Cash, 2012; Spencer et al., 2018). Such changes may adversely affect neuropsychological function (Fisher et al., 2005). Subjects with temporal lobe epilepsy (TLE) often experience cognitive impairment in different domains including memory (Ozkara et al., 2004), language (Kaestner et al., 2019), attention (Celiker Uslu et al., 2019), and executive function (Oyegbile et al., 2004, 2018), which may already be present from the moment of diagnosis (Pugh et al., 2024a, 2024b). In some patients, these cognitive comorbidities can be more disabling than the seizures themselves. Hence, the characterization of the cognitive profile of each patient from the moment of diagnosis and throughout the treatment is of vital importance (Mula et al., 2022). Despite its clinical importance, the neurobiological basis of cognitive dysfunction in epilepsy remains poorly understood.

### 1.1 The heterogeneous nature of cognitive impairment in temporal lobe epilepsy

Cognitive profiles in TLE are complex and heterogeneous. The high variability in cognitive impairment among patients, limited knowledge regarding etiological factors (anti-seizure medications, seizure type, etc.), and the absence of a continuous neuropsychological characterization from the beginning and throughout the treatment result in an incomplete understanding and inadequate management of cognitive and behavioral disorders in epilepsy. Impairments are often conceptualized within a framework of hemispheric specialization, where left TLE is typically associated with deficits in verbal memory and language, and right TLE with visuospatial and non-verbal memory impairments (Scoville and Correll, 1973). However, empirical evidence for this dissociation is mixed, with many patients exhibiting deficits that cross these traditional boundaries (Rice et al., 2018).

### 1.2 Advances and inconsistencies in connectivity research

Over the past decade, structural and functional neuroimaging have begun to shed light on the mechanisms underlying cognitive deficits in TLE. Task-based fMRI studies have exposed abnormalities of neural activity in specific regions associated with particular cognitive domains (Oyegbile et al., 2019; Fleury et al., 2022; Peng et al., 2023; Fajardo-Valdez et al., 2024). Structural and functional studies at rest have also shown abnormalities in various networks that go beyond the temporal lobe region (Doucet et al., 2016; Yang et al., 2018a; Chang et al., 2019). Studies examining both static and functional connectivity (Pang et al., 2022; Song et al., 2025) found that abnormal dynamic network properties correlate with poorer performance in cognitive domains such as language, conceptual thinking, and memory.

More recently, a few studies have addressed the complex relationship between multivariate patterns of brain connectivity and specific cognitive deficits in epilepsy (Fang et al., 2015; Sajobi et al., 2017; Rodríguez-Cruces et al., 2020). These studies provide multivariate evidence that the topology of white matter networks is closely linked to the heterogeneous cognitive profiles observed in TLE. Specifically, they demonstrate that reduced efficiency and altered hub connectivity are strongly associated with the degree and pattern of cognitive impairment. This underscores the value of connectome-level analyses in understanding and potentially predicting cognitive outcomes in epilepsy. Furthermore, Fang et al. (2015) revealed that left and right TLE exhibit distinct patterns of anatomical connectivity disruption, especially within cortico-limbic and cerebellar networks.

Despite these advances, the field is marked by significant inconsistencies. For instance, cognitive impairment in TLE has been associated with both increases and, conversely, decreases in connectivity within specific networks (Ives-Deliperi and Butler, 2021; Yang et al., 2018b; Khalife et al., 2022) as well as in global network properties (van Diessen et al., 2014; Slinger et al., 2022). These contradictions largely reflect methodological heterogeneity in neuroimaging protocols, network definitions, and statistical thresholds. Similar inconsistencies exist in cognitive profiles, where studies often differ in the domains assessed or simply dichotomize patients (impaired vs. unimpaired) without characterizing the nature of the deficit. Consequently, there is a critical gap in integrating these advanced connectivity metrics with precise, multidimensional, and quantitative behavioral phenotyping.

### 1.3 Robotic technology for objective cognitive phenotyping

Traditional neuropsychological batteries may be time-consuming, require specialised administration, and often yield limited quantitative outcomes. Robotic tools have been shown to provide rapid and accurate assessments of cognitive impairment (Scott and Dukelow, 2011; Scott et al., 2023). The reliability of the Kinesiological Instrument for Normal and Altered Reaching Movements (Kinarm) has been demonstrated for the evaluation of sensory, motor and cognitive function in healthy people and in different disorders (Mang et al., 2018; Simmatis et al., 2019, 2020a). Recently, the feasibility and validity of Kinarm robotic assessment has been demonstrated in individuals with epilepsy (Finn et al., 2024; Aliyianis et al., 2026; Simmatis et al., 2020b).

### 1.4 Research gap and objectives

To date, no study has combined the objective, quantitative cognitive profiles generated by robotic assessment with comprehensive, multivariate models of brain network connectivity in TLE. To address this, the present study combines Kinarm robotic phenotyping with connectome-based neuroimaging to test the hypothesis that specific large-scale network alterations underlie domain-specific cognitive impairments in TLE. This approach moves beyond descriptive associations toward an integrated, mechanistic understanding of the neural substrates of cognitive heterogeneity in TLE, paving the way for personalized treatments that target cognitive comorbidities.

## 2 Methods

### 2.1 Participants

The participants were prospectively recruited from epilepsy clinics at Kingston Health Sciences Centre, Ontario, Canada. Participant inclusion was based on diagnosis of TLE by an epileptologist (GS, LBL, GPW) in accordance with the International League Against Epilepsy guidelines, relying on clinical semiology, routine EEG, and conventional neuroimaging. Only subjects within the age range of 18-65 years old were included. Exclusion criteria included neurodegenerative disease, significant intellectual or physical impairment (causing inability to perform the cognitive and/or Kinarm tasks), inability to speak and/or understand English and previous neurosurgery. Healthy controls with no history of psychiatric or neurological disorders were also recruited for the study. All participants provided written informed consent prior to study participation, in line with the Declaration of Helsinki and as approved by the Queen's University Health Sciences and Affiliated Teaching Hospitals Research Ethics Board (DMED-2390-20).

### 2.2 Robotic cognitive screening

The cognitive screening was performed using the Kinarm End-Point Lab developed at Queen's University (Scott, 1999). Kinarm provides objective parameters of motor, sensory, and cognitive function based on a series of upper limb behavioural task. All participants performed nine tasks: Visually Guided Reaching (VGR) (Coderre et al., 2010; Mostafavi et al., 2014), Reverse Visually Guided Reaching (RVGR) (Hawkins and Sergio, 2014), Object Hit (OH) (Tyryshkin et al., 2014), Object Hit & Avoid (OHA) (Bourke et al., 2016), Trail Making A (TMA) (Tombaugh, 2004; Bowie and Harvey, 2006), Trail Making B (TMB) (Bowie and Harvey, 2006), Paired-Associates Learning (PAL), Ball on Bar (BOB) (Lowrey et al., 2014) and Arm Position Matching (APM) (Dukelow et al., 2010). Kinarm tasks quantify various aspects of behaviour across a set of task parameters where performance is scored relative to a standard, normal model of performance for healthy individuals that consider the influence of sex, age and handedness (Scott et al., 2023; BKIN Technologies Ltd. d.b.a. Kinarm, 2023). The normative datasets include 100 to 1000 participants in a given task, and were collected at research labs at Queen's University and Kingston-area hospitals (Kingston Health Sciences Centre, Providence Care Hospital and St. Mary's On The Lake Hospital). An aggregate score is also provided that combines these task parameters as a global measure of a subject's performance for a given task. This global measure called Z-*Task Score* is generated using the root-mean-square of the task parameter z-scores, and again, scores performance relative to a standard, normal model for healthy individuals. Z-Task Score was used for all statistical tests and analyses. For graphical purposes, a Task Score was also calculated obtaining a one-sided measure, such that a Task Score of 0 is the best possible performance, with higher positive scores indicating worse performance (for details of each task and normative datasets, see Supplementary Table S1 or https://Kinarm.com/solutions/Kinarm-standard-tests/.

### 2.3 MRI acquisition, preprocessing and connectivity estimation

All images were obtained with a 3-T Siemens Magnetom Prisma scanner located in the Centre for Neuroscience Studies at Queen's University. The sequences used were adapted from established protocols in the Human Connectome Project (http://www.humanconnectomeproject.org/). For detailed MRI acquisition parameters, see Supplementary Table S2.

The open-access pipeline micapipe (v0.2.2 http://micapipe.readthedocs.io/) (Cruces et al., 2022) was used for structural connectivity calculation. Briefly, the T1-weighted images were de-obliqued, reoriented, intensity non-uniformity corrected, and skull stripped. DWI data were denoised, corrected for susceptibility distortions, head motion, and eddy currents. Tractography was performed using a probabilistic approach with 10 million streamlines (Tournier et al., 2012). The connections weights were defined as the SIFT (Yeh et al., 2016) weighted number of streamlines between regions. This pipeline incorporates an integrated quality control module that produces both individual- and group-level reports. This module evaluates DWI preprocessing steps, with a focus on detecting registration failures and imaging artifacts to ensure data quality prior to tractography.

The rs-fMRI data were preprocessed with the fMRIPrep pipeline (Esteban et al., 2019) including removal of the first five volumes, image re-orientation, motion and distortion correction. For each subject, the detailed fMRIPrep quality control report was manually inspected to confirm preprocessing adequacy. Specific checks included anatomical-to-functional alignment, registration accuracy, brain extraction quality, and quantitative motion parameter analysis. For nuisance regression white matter and CSF signal (plus their temporal derivatives and quadratic terms), low-frequency temporal regressor for high-pass temporal filtering, and 24 motion regressors (6 base motion parameters + 6 temporal derivatives + 12 quadratic terms) were used. Individual functional connectomes were generated by cross correlating all nodal time series.

For the present study, a total of 116 nodes were defined merging the cortical parcellation from the Schaefer atlas (100 nodes) (Destrieux et al., 2010) and subcortical FreeSurfer segmentation (Fischl et al., 2002).

### 2.4 Dimensionality reduction

**Fig. 1:**
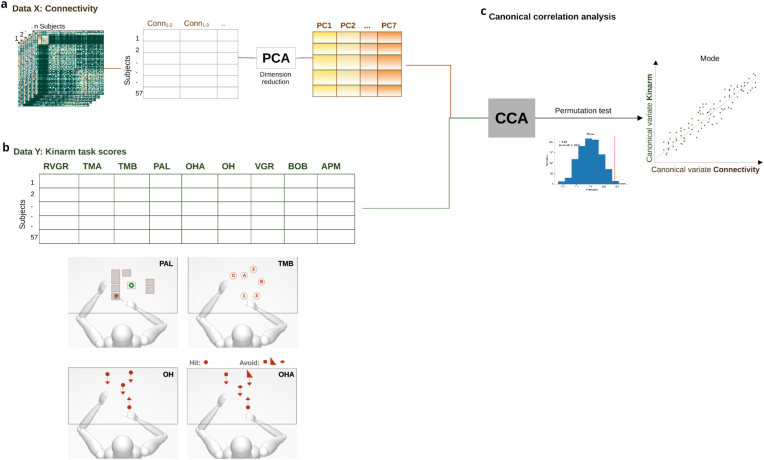
Scheme of multivariate brain-behavior associations analysis. **a**) The 116 x 116 connectivity matrices was obtained for each subject and flattened to obtain one matrix of connectivity features per subject. The connectivity data was transformed into principal components before the CCA model evaluation. **b**) Normalized behaviour data measured by Kinarm screening. **c**) Each connectivity and behavioural matrices were entered into the CCA analysis. In the scatter plot each subject can be described by two canonical variates maximally correlated per mode. The significant mode was determined by permutation test.

Principal Component Analysis (PCA) was used to reduce the high dimensionality of the SC and FC into principal components (PCs) identifying the dominant patterns of regional (Fig. 1a). A total of 6670 connectivity features for each participant were obtained after flattening the upper triangle of the 116 x 116 connectivity matrix. Connectivity values were residualized to ensure the age, sex, and level of education did not drive the results. To determine the optimal number of Pcs, we employed a two-step criterion: 1) eigenvalue threshold: components with eigenvalues equal to or greater than 1 (Guttman-Kaiser rule) were retained, ensuring each component explains at least as much variance as one standarized original feature; 2) stability validation to verify robustness against sampling variation: 90% of the sample was randomly selected for the PCA estimation and repeating the procedure 5000 times. This combined approach consistently identified seven stable components for both SC and FC (using Python scikit-learn v1.3.0). The selected components accounted for 52% of the total variance in SC and 72% in FC.

### 2.5 Data analysis

#### 2.5.1 Demographic and cognitive group comparisons

Data normality was assessed using the Shapiro-Wilk test. Based on this assessment, group differences in demographic and cognitive variables between individuals with TLE and healthy controls were tested using Mann-Whitney *U* test (for non-normally distributed data) or a Student's t-test (for normally distributed data).

#### 2.5.2 Sparse Canonical Correlation Analysis (sCCA)

Sparse canonical correlation analyses (sCCA) were used to delineate the maximal correlations between linear combinations of variables in connectivity and behaviour data, with regularization to achieve sparsity (Fig. 1c). sCCA imposes both L1-norm and L2-norm penalty terms, an elastic net regularization combining the Lasso and Ridge penalties. This regularization promotes a sparse solution, enhancing the interpretability of the resulting variates by selecting a subset of driving features.

#### 2.5.3 Model tuning and statistical inference

Parameter selection: The optimal penalty parameters (one for the connectivity and one for the behavioral variable set) were selected via 3-fold cross validation (k = 3) combined with a grid search. The search space spanned the interval [0, 1] for each parameter in increments of 0.1.

Significance testing: The statistical significance of the resulting canonical correlations was assessed using a permutation test. The behavioral data were randomly shuffled 5000 times, with the entire sCCA model (including parameter tuning) re-run for each permutation to generate a null distribution of correlation strengths. A canonical mode was considered significant if its correlation coefficient exceeded the 95th percentile of this null distribution (p < 0.05).

Stability and generalizability: To estimate the out-of-sample reliability and generalizability of the significant associations, we performed a 3-fold cross-validation procedure repeated 1000 times. In each iteration, the inner tuning and model fitting were performed on a training set (2/3 of the data), and the canonical correlation was computed on the held-out test fold (1/3 of the data). The distribution of test-fold correlations across all iterations provides a robust performance estimate.

#### 2.5.4 Interpretation of significant canonical modes

Each canonical mode represented a distinct pattern that relates a weighted set of cognitive features to a weighted set of brain connections. After identifying statistically significant canonical modes, we inspected the most heavily weighted features for each mode using Pearson's correlation between the variate score and the original variable. This allows us to identify which Kinarm tests and which brain connections have more weight in driving the significant association. Specifically, the correlation between the Kinarm canonical variate and the Kinarm score (original variable) identified the cognitive domains most heavily weighted in each CCA mode. Similarly, the correlation between the connectivity canonical variate and the connectivity metric before being reduced into principal components (original variable) revealed which connectivity features most strongly drive the multivariate relationship. A similar PCA-CCA framework has been extensively applied in studies of brain-behavior associations (Smith et al., 2015; Mihalik et al., 2022).

#### 2.5.5 Assessing the influence of covariates

To evaluate whether the identified brain-behavior relationships were confounded by or independent of key clinical/demographic factors, we conducted supplementary analyses. The covariates included (education level, epilepsy duration) were selected based on data availability and reliability. First, we assessed the simple associations of the cognitive measures (Kinarm score) with education level and duration of epilepsy using Spearman correlation. Second, to directly test the influence of these covariates on the canonical associations, we performed a multiple regression analysis. In this model, the connectivity canonical variate for each significant mode was regressed onto the corresponding cognitive canonical variate, with education level and duration of epilepsy included as simultaneous predictors. This allowed us to determine whether the brain-behavior relationship remained significant after accounting for these potential confounders.

## 3 Results

### 3.1 Sample characteristic

A total of fifty-seven participants were recruited, including thirty-three individuals with TLE (median of age 34, IQR range 29–43 years, 16 female) and twenty-four healthy controls (median of age 29, IQR range 23–39 years, 18 female). Among the patient cohort, 22/33 (67%) subjects had left TLE, 10/33 (30%) had right TLE and 1/33 (3%) patient had bilateral TLE. There were no statistically significant differences between groups in the distribution of gender and handedness, but controls tended to be younger although not statistically significant (p = 0.053) and had a greater number of years of education (Table 1).

**Table 1:** Sample characteristics.

	Controls (N = 24)	TLE (N = 33)	Statistic	p-value
Median age (IQR)	29 (23 - 39)	34 (29 - 43)	U = 276	0.053
Age range	19 - 56	20 - 61	-	-
Sex (F/M)	18/6	16/17	χ^2^ = 3.03	0.08
Median years of education (IQR)	16 (14 - 18)	12 (12 - 14)	U = 609	<0.01
Hand (L/R)	2/22	3/30	χ^2^ = 0	1.0
Duration of epilepsy (years)	-	9.58 (±11.04)	-	-

### 3.2 Robotic cognitive profiles

**Fig. 2:**
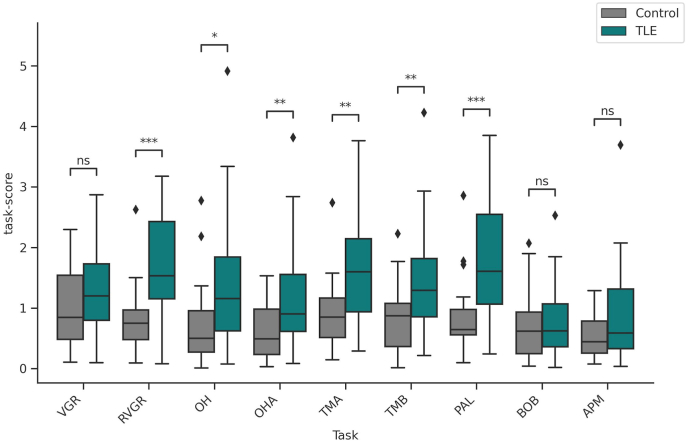
Kinarm screening. Boxplot of the of Kinarm Task scores across each task. VGR: Visually Guided Reaching. RVGR: Reverse Visually Guided Reaching. OH: Object Hit. OHA: Object Hit & Avoid. TMA: Trail Making A. TMB: Trail Making B. PAL: Paired-Associates Learning. BOB: Ball on Bar. APM: Arm Position Matching. ∗: 1e-02 < p≤5e-02. ∗∗: 1e-03 < p≤1e-02. ∗∗∗: 1e-04 < p≤1e-03. ∗∗∗∗: p≤1e-04, ns: 5e-2 < p≤1.

Compared to our controls, patients had significantly worse performance in almost all tasks (Table 2), including RVGR for both hands (*p* < 0.0001), PAL (p = 0.0003), TMA (p = 0.001), TMB (p = 0.005), OHA (p = 0.006), and OH (p = 0.01). Fig. 2 displays Kinarm scores per task comparison between control and TLE groups across each task.

**Table 2:** Summary of data for Kinarm Task scores.

Task	Controls (N = 24) Median (IQR)	TLE (N = 33) Median (IQR)	Statistic	p-value	Effect size (r)
VGR	0.84 (0.48 – 1.54)	1.2 (0.8 – 1.73)	309	0.16	0.22
RVGR	0.75 (0.48 – 0.97)	1.53 (1.15 – 2.43)	156	0.0001	0.61
OH	0.5 (0.27 – 0.95)	1.16 (0.62 – 1.84)	237	0.01	0.4
OHA	0.49 (0.23 – 0.98)	0.9 (0.61 – 1.55)	232	0.008	0.41
TMA	0.85 (0.51 – 1.17)	1.6 (0.94 – 2.14)	199	0.001	0.5
TMB	0.87 (0.37 – 1.07)	1.29 (0.85 – 1.82)	228	0.007	0.42
PAL	0.64 (0.56 – 0.98)	1.6 (1.07 – 2.55)	172	0.0003	0.57
BOB	0.62 (0.25 – 0.93)	0.63 (0.36 – 1.07)	376	0.75	0.05
APM	0.44 (0.26 – 0.78)	0.59 (0.33 – 1.32)	326	0.26	0.18

Four tasks (OH/OHA/TMB/APM) were significantly correlated with educational level, with higher-educated subjects performing better on these tasks (Table 3). There were no significant relationships between any of the Kinarm tasks and the duration of epilepsy (Table 3).

**Table 3:** Correlation between Kinarm scores and educational level and duration of epilepsy.

Task	Educational level	Duration of epilepsy
VGR	−0.35	−0.08
RVGR	0.08	0.28
OH	−0.58∗∗∗	−0.26
OHA	−0.58∗∗∗	−0.21
TMA	−0.32	0.09
TMB	−0.47∗∗	0.19
PAL	−0.28	−0.02
BOB	−0.22	0.07
APM	−0.45∗∗	−0.008

∗∗1e-03 < p≤1e-02. ∗∗∗: 1e-04 < p≤1e-03.

### 3.3 Connectivity associated with robotic assessment

#### 3.3.1 Structural connectivity (SC) patterns by cognitive domain

**Fig. 3:**
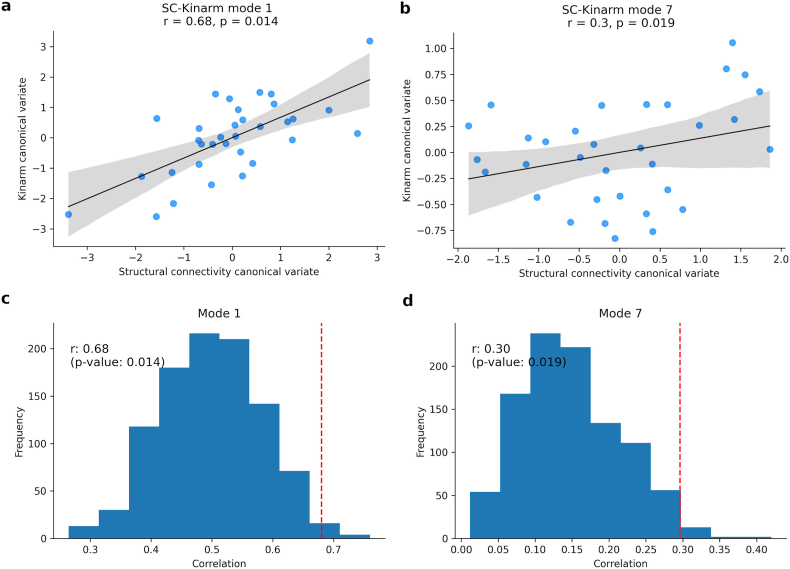
Multivariate patterns of robotic cognitive assessment and structural connectivity (SC). Scatter plot showing the correlation **a**) for the first significant mode (r = 0.68, p = 0.014) and **b**) for a second significant mode (r = 0.3, p = 0.02), where each dot represents an individual subject. **c-d**) The null distribution of canonical correlation with randomly shuffled data (histogram) and the true canonical correlation (red dashed line) for each significant mode.

We found two significant modes of covariation between patterns of SC and sets of Kinarm scores across participants with TLE (Fig. 3).

**Fig. 4:**
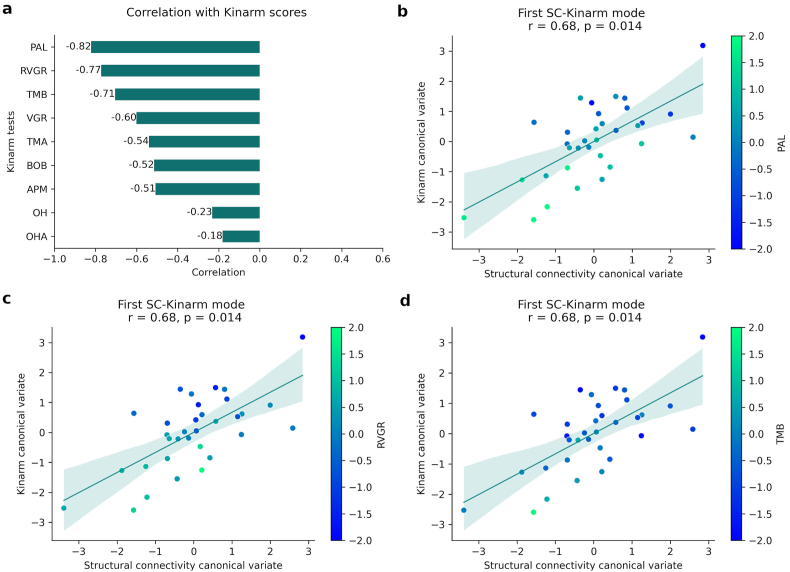
**a**) Correlation between the original Kinarm task-score and Kinarm canonical variate of the first mode (r = 0.68, p = 0.014). Panels b-d show the same canonical variate scores for each participant, coloured by: **b**) PAL scores, which is the Kinarm task that contributed the most to this canonical variate (r = −0.82); **c**) RVGR scores (r = −0.77) and, **d**) TMB scores (r = −0.71).

**Memory and executive function**. The correlation between the original variables, Kinarm scores and connectivity values, and the canonical variates identified the cognitive domains and brain connections most strongly associated with each CCA mode. This mode was primarily defined by performance on the PAL (visual/episodic memory (Barnett et al., 2016)), RVGR, and TMB (inhibitory control, working memory) tasks (Fig. 4a). In these tasks, subjects with lower task-scores (better performance) clustering towards higher canonical scores and subjects with higher task-scores (worse performance) clustering towards lower canonical scores (Fig. 4b–d).

**Fig. 5:**
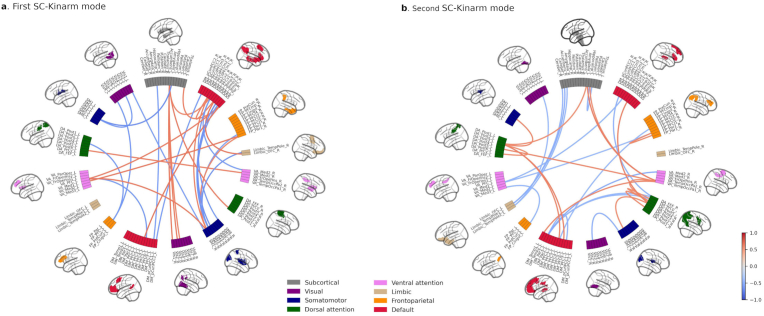
Correlations between SC variables and SC canonical variate of the a) first and b) second significant CCA modes. Only top 15 most positively (red connections) and top 15 most negatively (blue connections) correlations are shown. Nodes are colour coded by resting state networks assigning each node to one of the seven cortical networks described in Yeo et al. and subcortical segmentation (Destrieux et al., 2010).

The underlying structural network involved widespread integration across major systems (Fig. 5a). Better performance was associated with stronger connectivity (negative correlations) in pathways linking the somatomotor and limbic networks (e.g., SMN-amygdala), the default mode network (DMN) with attention and visual regions (e.g., precuneus-visual network), and the hippocampus with the DMN (blue edges in Fig. 5a). Conversely, poorer performance was linked to stronger connectivity (positive correlations) in a separate set of pathways, particularly involving inter-hemispheric connections between attention, frontoparietal, and subcortical networks (red edges in Fig. 5a). The list of 15 structural connections most negatively/positively associated with this SC-cognitive mode are described in Supplementary Table S3/S4. We have selected the threshold of 15 for better visualization after checking that the patterns were consistent across different connection strength thresholds (Figs. S1 and S2).

**Fig. 6:**
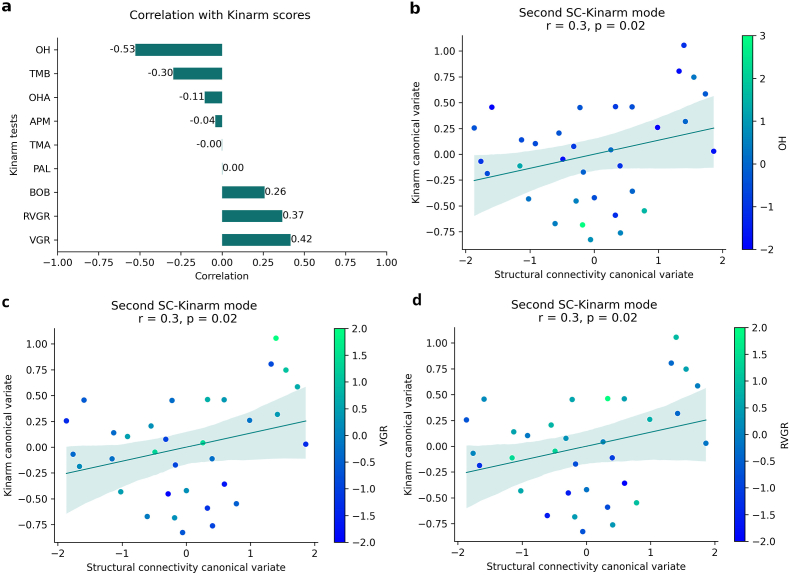
**a**) Correlation between the original Kinarm task-score and Kinarm canonical variate of the second significant mode (r = 0.3, p = 0.02). Scatter plot showing the correlation for this mode where each dot represents an individual subject and coloured by: **b**) OH scores, which is the Kinarm task that contributed the most to this canonical variate (r = −0.53); **c**) VGR scores (r = 0.42) and, **d**) RVGR scores (r = 0.37).

**Sensorimotor performance**. The second significant CCA mode (SC-Kinarm mode 2, r = 0.30, p = 0.02) captured a sensorimotor component (Fig. 6). Specifically, OH scores, which primarily evaluate visuomotor skills, had the highest correlation (r = −0.53) with the Kinarm canonical variate, followed by the TMB task (r = −0.3) (Fig. 6a). In both tasks, individuals with TLE presented a worse performance. In this case, subjects with lower OH scores (better performance) had higher Kinarm canonical scores and vice versa (Fig. 6b), but without observing similar behavior with the SC canonical score. On the other hand, VGR and RVGR correlated positively, although to a lesser extent (r = 0.42 and r = 0.37, respectively). Individuals with better performance tend to present lower Kinarm canonical variate values (Fig. 6c and d). Considering that the highest correlation was with OH, followed by VGR, two tests that evaluate motor control and visuospatial processing, we can say that this second connectivity-cognitive mode captures mainly the sensorimotor component.

The structural signature of this mode was characterized by intra-hemispheric connections primarily within the dorsal and ventral attention networks (DAN/VAN), linking them to the somatomotor network, prefrontal cortex, and limbic structures (Fig. 5b). The list of 15 structural connections most positively/negatively associated with this SC-Kinarm mode are described in Supplementary Table S5/Table S6.

#### 3.3.2 Functional connectivity (FC) pattern

**Fig. 7:**
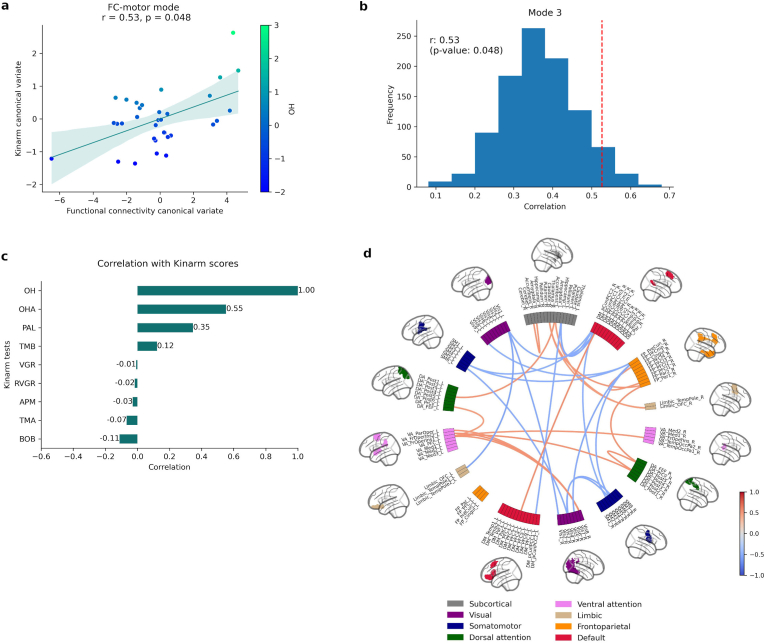
Multivariate pattern of Kinarm screening and functional connectivity (FC). **a**) Scatter plot showing the overall correlation (r = 0.53, p = 0.048) between FC and Kinarm scores, where each dot represents an individual subject. Subjects are colour coded by OH task scores. **b**) The null distribution of canonical correlation with randomly shuffled data (histogram) and the true canonical correlation (red dashed line). **c**) Correlations between each original Kinarm task and Kinarm canonical variate. **d**) Correlations between FC variables and FC canonical variate. Only top 15 most positively (red edges) and top 15 most negatively (blue edges) correlations are shown. Nodes are colour coded by resting state networks assigning each node to one of the seven cortical networks described in Yeo et al. and subcortical segmentation (Destrieux et al., 2010).

Only a single mode of covariation between FC and Kinarm screening was significant across participants with TLE, yielding a canonical correlation of r = 0.53 (p = 0.048) (Fig. 7a). This FC mode was most strongly associated with the sensorimotor domain. Fig. 7c shows the correlation of each original Kinarm task score with Kinarm canonical covariate, where OH was the test with the maximum correlation (r = 1), followed by OHA (r = 0.55), with subjects with worse performance clustering towards higher scores and subjects with better performance clustering towards lower scores. While OH primarily evaluates motor skills and attention, OHA also examines rapid decision-making and motor inhibition. The most strongly associations between FC variables and FC canonical variate were negative (blue edges in Fig. 7d) included connections between precuneus and visual network, SMN and hippocampus. Functional connections most positively correlated (red edges in Fig. 7d) included a sensorimotor-attention-limbic circuit, particularly involving the ventral attention network (frontal operculum/insula), dorsal attention network, and connections to the caudate and amygdala. The list of 15 structural connections most negatively/positively associated with the FC-Kinarm mode are described in Supplementary Table S7/Table S8.

#### 3.3.3 Integration of SC-FC findings

The significant CCA modes show different but complementary findings from SC and FC related to robotic cognitive assessment in TLE. SC analysis found two distinct modes: one linked to memory and executive functions, the other to sensorimotor abilities. In contrast, FC analysis identified a single mode that maps onto the sensorimotor domain. Although both modalities converge on sensorimotor-related networks, they highlight different aspects of the underlying neurobiology. The SC signature (mode 2), which involves intra-hemispheric pathways within and between attention, somatomotor, and prefrontal regions, emphasizing the anatomical backbone that supports cognition. The FC signature, on the other hand, captures the dynamic interplay within a sensorimotor-attention-limbic circuit, particularly involving the ventral attention network, amygdala, and caudate.

## 4 Discussion

The main contribution of this work is the identification of multivariate associations between brain connectivity patterns, both structural determined by DWI and functional determined by rs-fMRI, and cognitive impairment detected by robotic screening in subjects with TLE. These results support previous work demonstrating the potential for connectivity-based dimensions of cognitive deficits and add novelty in using Kinarm technology. To the best of our knowledge, this is the first study to investigate the structural-functional underpinnings of cognitive and sensorimotor impairment detected by robotic testing in individuals with TLE. Given the exploratory nature of this integration and our modest sample size, these findings should be interpreted as providing preliminary, proof-of-concept evidence that require replication and validation in larger, independent cohorts.

### 4.1 Robotic cognitive profiling in TLE

Kinarm robotic screening revealed a distinct pattern of cognitive impairment in individuals with TLE compared to controls. While deficits were most prominent in memory and executive function, alterations were also observed in some components of the perceptual-motor domain. The impairment was reflected in all the tests that evaluate different aspects of executive function and memory (RVGR/PAL/TMA/TMB/OHA) and in only two tests that evaluate some components of the perceptual-motor domain (TMA/OH). No significant differences were found in the rest of the tasks that examine this domain (VGR/BOB/APM). This pattern was particularly evident in the RVGR and PAL tasks, which showed the higher effect sizes (Table 2). The selective impairment on RVGR, but not VGR, points to a specific deficit in the cognitive aspects of motor and cognitive integration. Similarly, impaired PAL performance suggests compromised visual and episodic memory. Further evidence of cognitive dysfunction emerged from performance on TMA and TMB, indicating impairments in processing speed, complex attention, and executive functions such as inhibition, working memory, and switching (Llinàs-Reglà et al., 2017). Finally, the TLE group also exhibited poorer performance on the OH and OHA tasks, suggesting deficits in rapid visuomotor/visuospatial skills and the cognitive processes required for rapid motor planning (Tyryshkin et al., 2014; Bourke et al., 2016). Taken together, these findings highlight a profile of cognitive impairment in TLE characterized by prominent deficits in memory, executive function, and attention, accompanied by less severe but notable impairments in perceptual-motor abilities.

The Kinarm robot is a relatively novel tool, with a few studies utilizing it in the neuropsychological assessment of individuals with epilepsy (Finn et al., 2024; Aliyianis et al., 2023; Simmatis et al., 2020b; Aliyianis, 2023; Falah, 2024). These studies have also found cognitive and sensorimotor impairments, including processing speed using a novel Kinarm test (Falah, 2024). Two investigations explored the relationship between the Kinarm test and the standard neuropsychological assessments (pen and paper) (Finn et al., 2024; Aliyianis, 2023), finding a significant correlation between PAL and RBANS Delayed Memory and Immediate Memory (Randolph et al., 1998), also between OH/OHA and RBANS Attention, and between the Trail Making tests examined by both techniques. However, more extensive validation against a comprehensive set of conventional neuropsychological tests is needed to fully establish the convergent and discriminant validity of robotic metrics and to define their specific clinical utility.

### 4.2 Translational relevance of robotic assessment

This work helps to establish the framework for integrating Kinarm into clinical routine. Robotic screening with Kinarm offers the opportunity for a comprehensive, detailed, and feasible automated assessment within the clinical setting, requiring less time and operator cost in comparison with traditional neuropsychological evaluation. Also, robotic assessment and scoring are fully automated, ensuring high objectivity and minimizing examiner bias. Standard testing requires trained examiners, and scoring can involve a degree of subjectivity. The translational relevance of our findings lies in this complementary role. By linking specific kinematic deficits (e.g., OH performance) to distinct brain network alterations, we move toward a precision phenotyping model. This knowledge paves the way for targeted interventions—such as cognitive rehabilitation focused on specific neural circuits or neurofeedback protocols—and provides objective, repeatable biomarkers for longitudinal monitoring of disease progression or treatment response (e.g., pre-*vs*. post-surgical, medication changes).

### 4.3 Neural substrates of robotic performance

Two distinct patterns of structural connectivity were found to be associated with domain-specific cognitive performance in the TLE cohort. The first, characterized by intra-hemispheric connections within the right hemisphere (between SMN and DMN in temporal and ventral PFC, the temporal pole of the limbic system, and parietal FPN) and within the left hemisphere (between the precuneus, posterior DAN, and visual network), was linked to worse performance on PAL, RVGR, and TMB tasks (Fig. 5, Fig. 4b-d). The second, involving inter-hemispheric connections (primarily between left PFC and right parietal DMN and SMN, left nucleus accumbens and visual network, and right posterior DMN and ventral PFC), was associated with better performance on these tasks. Notably, both patterns were consistent across different connection strength thresholds (Fig. S1). Since PAL, RVGR, and TMB primarily assess memory and executive functions, these findings highlight the role of distinct intra- and inter-hemispheric connectivity patterns in modulating memory and executive function performance in individuals with TLE. The specificity of these structural connectivity associations is a key finding; however, the more limited FC results suggest functional network relationships may be more diffuse or variable in this cohort.

Notably, performance on the Object Hit (OH) test was associated with both the second SC-Kinarm mode and the FC-Kinarm mode. The OH task requires rapid visuomotor and visuospatial skills, including goal-directed motor actions, bimanual planning, attention, motor speed, and visuomotor control (Simmatis et al., 2020b). Crucially, impairment on this task, as identified by our robotic assessment, provides an objective correlate for the attention deficits, slower reaction times, and reduced psychomotor speed widely reported in TLE (Zheng et al., 2000; Piazzini et al., 2006; Bocquillon et al., 2008; Sung et al., 2013). The findings suggest a compelling dissociation in the neural underpinnings of these deficits. The second SC-Kinarm mode highlighted an intra-hemispheric structural connectivity pattern primarily within attentional networks (posterior DAN, PFC and VAN).In contrast, the FC-Kinarm mode revealed a functional connectivity pattern involving regions critical for visuomotor integration, including the right precuneus (implicated in visuospatial navigation (Duan et al., 2025)) and the left hippocampus (involved in motor learning (Gheysen et al., 2010)). This suggests that while structural connectivity alterations within core attention networks are involved, impaired functional integration across a wider visuomotor network also contributes to performance. To our knowledge, this is the first report to link deficits in visuospatial attention and visuomotor control in TLE to distinct patterns of structural and functional brain connectivity, potentially reflecting the integration of visual, spatial, and memory processes. Importantly, these connectivity patterns remained stable across different thresholds for defining top connections (Figs. S1 and S2).

The present findings both reinforce previously reported evidence and offer novel insights into the neural underpinnings of cognitive impairment in TLE. The first SC-Kinarm mode highlighted a pattern implicating the DMN, amygdala, hippocampus, and precuneus -- regions consistently linked to memory and executive function deficits (O'Shea et al., 2016; Kumral et al., 2021; Ballerini et al., 2023). Alterations in the connectivity of these regions have been well-documented in individuals with TLE (Riley et al., 2010; Liao et al., 2011; Legouhy et al., 2023; Zanao et al., 2023; DeSalvo et al., 2014; Voets et al., 2012), and prior work has specifically associated functional and structural connectivity changes within the amygdala and hippocampus with memory and executive dysfunction in this population (Stretton et al., 2013; Dinkelacker et al., 2015; Qin et al., 2020). However, the specific architecture of inter- and intra-hemispheric white matter connectivity underlying these epilepsy-related cognitive changes remains less clear. This study identified distinct intra-hemispheric structural connectivity patterns associated with cognitive impairment, potentially reflecting alterations in the functional specialization of different cognitive functions, as suggested by previous research on hemispheric white matter connectivity (Iturria-Medina et al., 1991; Caeyenberghs and Leemans, 2014; Friedrich et al., 2022). For instance, the involvement of the posterior parietal cortex, a key component of the DAN implicated in visuospatial attention and sensorimotor integration (Berndt et al., 2019; Hadjidimitrakis et al., 2019), suggests that disruptions within this network may contribute to the observed cognitive deficits.

The second SC-Kinarm mode and the FC-Kinarm mode both captured connectivity patterns associated with sensorimotor dysfunction, and notably, performance on OH task. The SC-Kinarm mode revealed an intra-hemispheric structural connectivity pattern involving the posterior parietal cortex, correlating with performance on tasks requiring perceptual-motor skills and attention. Further, the FC between this region and the precentral ventral DAN, as well as the frontal operculum/insula of the VAN, was also associated with OH performance. Given the VAN's role in bottom-up attention to unexpected stimuli (Vossel et al., 2014) and the precentral ventral DAN's involvement in voluntary eye movements and top-down attentional control (Vossel et al., 2014), these findings suggest that altered connectivity within these attention networks may underlie the observed visuomotor deficits. Interestingly, while the SC-Kinarm mode showed a negative correlation with OH performance, the FC-Kinarm mode showed a positive correlation, primarily within attention networks. This positive correlation might reflect reduced functional specialization or segregation within these networks. Indeed, abnormal functional and metabolic connectivity within the DAN, VAN, and DMN have been previously reported in epilepsy (Burianová et al., 2017; Jiang et al., 2018; Zhou et al., 2020; Bacon et al., 2023; Wang et al., 2023; Zhang et al., 2024) and linked to attentional deficits (Zhou et al., 2020; Zhang et al., 2009; Killory et al., 2011).

### 4.4 Consideration of confounds

We examined if education level and epilepsy duration could confound the observed relationship between brain connectivity and robotic cognitive profiles. We found that epilepsy duration showed no significant association with overall cognitive performance (Table 3, a result aligning with previous works based on Kinarm screening (Finn et al., 2024; Aliyianis, 2023) and with evidence that cognitive deficits can be present at diagnosis (Pugh et al., 2024b; Jackson-Tarlton et al., 2020; Bjørke et al., 2021). Lower education level, however, was associated with poorer performance on specific tasks OH/OHA/TMB/APM tasks. This is consistent with the high educational level role as cognitive reserve factor (Wang et al., 2020; Hernández et al., 2024). Crucially, neither variables significantly moderated the association between connectivity patterns and Kinarm cognitive profiles (see Tables S9 and S10), indicating that our primary findings are not artifacts of these variables.

### 4.5 Limitations and future directions

While this study provides valuable insights into the relationship between brain connectivity and cognition in TLE, certain limitations should be acknowledged. The modest sample size is a critical factor, particularly for multivariate approaches, and necessitates that these findings be confirmed in a larger, independent cohort to ensure reproducibility and generalizability.

Furthermore, our study could not fully account for several key clinical confounds. Although we controlled for education and epilepsy duration, other factors known to influence cognition and brain networks, such as anti-seizure medication load, seizure frequency, and psychiatric comorbidities (e.g., depression or anxiety) (Zhang et al., 2024; Reyes et al., 2019; Rodriguez-Cruces et al., 2022; Garcia-Ramos et al., 2023; Bingaman et al., 2023) were not included in our models. For anti-seizure medications, the large number of combinations of medications and dosages in patients compared with the sample size prevents controlling for this variable. Participants were asked to self-report seizure frequency, such retrospective survey data are known to be unreliable (Blachut et al., 2015; Wong et al., 2025). Their potential confounding effects on the observed brain-behavior relationships remain an important consideration and a clear target for future prospective studies with comprehensive phenotyping.

Moreover, due to the small sample we could not perform subgroup analyses based on seizure-onset laterality (e.g., left vs. right TLE). Given that laterality can significantly influence neuropsychological profiles (e.g., verbal memory deficits in left TLE, visuospatial deficits in right TLE) (Elverman et al., 2019; Phuong et al., 2021; Cairós-González et al., 2024), this remains an important area for future research in larger cohorts. Furthermore, the diverse cognitive profiles observed in TLE, ranging from intact cognition to domain-specific or generalized impairment (Oyegbile et al., 2004; Hermann et al., 2007), suggest that distinct connectivity-cognition relationships may exist within different patient subgroups.

Future work should focus on large, well-characterized cohorts with comprehensive phenotyping. By systematically recording medication load, seizure frequency, psychiatric comorbidities, and other pertinent variables, investigators can control for confounding influences and define robust subgroups. Longitudinal designs will make it possible to determine whether baseline connectivity-cognitive profiles can predict long-term cognitive trajectories or therapeutic outcomes. Additionally, the robotic metrics must be rigorously validated against standard neuropsychological batteries in a variety of epilepsy subtypes. This integrative approach will provide the foundation for mechanism-driven monitoring and personalized interventions targeting the cognitive comorbidities of TLE.

## 5 Conclusions

This study provides novel evidence that multivariate patterns of structural and functional brain connectivity are significantly associated with cognitive impairment in TLE, as revealed through comprehensive robotic assessment. These findings underscore the power of combining multivariate methods with advanced technologies like Kinarm to elucidate the complex brain-behavior relationships in TLE. While further research in larger cohorts is needed, this work lays the groundwork for a deeper understanding of cognitive comorbidities in TLE and offers a pathway towards personalized interventions that target both seizure management and cognitive enhancement.

## CRediT authorship contribution statement

**Karla Batista Garcia-Ramo:** Writing – original draft, Visualization, Methodology, Formal analysis. **Spencer Finn:** Writing – review & editing, Investigation, Data curation. **Theodore S. Aliyianis:** Writing – review & editing, Investigation, Data curation. **Adam Falah:** Writing – review & editing, Investigation, Data curation. **Brooke C. Beattie:** Writing – review & editing, Investigation, Data curation. **Donald Brien:** Writing – review & editing, Investigation, Data curation. **Garima Shukla:** Writing – review & editing, Resources. **Lysa Boissé-Lomax:** Writing – review & editing, Resources. **Stephen H. Scott:** Writing – review & editing, Resources, Methodology. **Jason P. Gallivan:** Writing – review & editing, Resources, Methodology. **Gavin P. Winston:** Writing – review & editing, Supervision, Resources, Project administration, Methodology, Funding acquisition, Conceptualization.

## Declaration of competing interest

The authors declare the following financial interests/personal relationships which may be considered as potential competing interests: G.P.W. reports financial support was provided by Physicians’ Services Inc Foundation and University Hospitals Kingston Foundation. J.P.G. reports a relationship with Panaxium Research Inc that includes employment. S.H.S. is the Co-Founder and Chief Scientific Officer of BKIN Technologies (dba as Kinarm) that commercializes the Kinarm robot used in this study. The other authors declare that they have no known competing financial interests or personal relationships that could have appeared to influence the work reported in this paper.

## Data Availability

The data that has been used is confidential.
