# Supplementary material for: Brain connectivity signatures of cognitive impairment in temporal lobe epilepsy identified by robotic assessment
Source: Neuroimage Rep. 2026 Feb 25;6(1):100330. doi: 10.1016/j.ynirp.2026.100330 (PMC13080667; doi:10.1016/j.ynirp.2026.100330)
Supplement: Multimedia component 1 [file mmc1.docx]

**Supplementary Information**

**Tables**

Table S1. Description of Kinarm tasks

Table S2. MRI acquisition parameters

Table S3. Negative correlations between SC variable and SC canonical variate of the first significant mode (cognitive)

Table S4. Positive correlations between SC variable and SC canonical variate of the first significant mode (cognitive)

Table S5. Positive correlations between SC variables and SC canonical variate of the second significant mode (sensorimotor)

Table S6. Negative correlations between SC variables and SC canonical variate of the second significant mode (sensorimotor)

Table S7. Negative correlations between FC variables and FC canonical variate of the significant mode (sensorimotor)

Table S8. Positive correlations between FC variables and FC canonical variate of the significant mode (sensorimotor)

Table S9 Multiple regression model structural connectivity

Table S10 Multiple regression model functional connectivity

**Figures**

Figure S1 Mean correlations between structural networks for the CCA modes

Figure S2 Mean correlations between functional networks for the CCA mode

**Table S****1**. Description of Kinarm tasks

| **Task** | **Description** | **Domain** | **Reference** |
| --- | --- | --- | --- |
| *Visually Guided Reaching* | The subject is instructed to an initial central target. Once there, the subject is instructed to quickly and accurately move to a peripheral target that appears. This process is repeated a number of times both to explore the workspace and to measure variability in the subject’s responses | visuomotor capabilities and multi-joint coordination | *[1]* |
| *Reverse Visually Guided Reaching* | The subject reaches the initial central target and in doing so, the movement of the cursor representing hand position is mirror reversed relative to the central target (i.e. the visual feedback is reversed compared to the actual hand position). From there the task requires the subject to cognitively override the normal response to move the hand directly to a target and instead initiate a movement in the opposite direction | attention, inhibitory control and cognitive control of visuomotor skills | *[2]* |
| *Trail making A* | The subject must trace through a sequence of targets numbered 1 to 25 as quickly as possible | visual search and motor speed skills | *[3]* |
| *Trail making B* | The subject must trace through an alternating alpha-numeric sequence of targets 1-A-2-B etc. up to 13, again for a total of 25 targets | cognitive flexibility | *[3]* |
| *Paired Associates Learning* | Images are shown in spatial locations and then hidden. Upon presentation of an image, the subject must indicate the spatial location of the hidden image | visual learning and episodic memory |  |
| *Object Hit* | Virtual paddles appear at the subject’s fingertips. The subject is instructed to use these paddles to hit and push away balls that are moving randomly towards the subject from one of 10 locations. As the task proceeds the balls move at greater speeds and appear more often, making the task more difficult as time progresses | rapid sensorimotor decision and control test | *[4]* |
| *Object Hit and Avoid* | At the start of the task the subject is shown 2 target shapes which the subject is instructed to memorize as the only shapes to hit during the task; they are instructed to avoid all other shapes or distractors (of which there are 6) | spatial attention, rapid motor selection and inhibition control | *[5]* |
| *Ball on Bar* | During the task, a virtual bar is presented in between the subject’s hands and a virtual ball is placed on the bar. There are 4 target circles that are presented to the subject one at a time. The object of the task is to move the virtual ball on the bar into each presented target as quickly and accurately as possible. The task has three levels. On the first level the ball is fixed to the centre of the bar. On the second level the ball’s position on the bar is a function of the angle of the bar. On the third level the ball can move freely on the bar | bimanual coordination and visuomotor skills | *[6]* |
| *Arm Position Matching* | During the task the Kinarm robot will move one of the subject’s arms to a given position, and the subject is instructed to move their other arm to a mirror-matched position. vision of the subject’s arms is blocked so that the subject can only use somatosensation from the arm to perceive arm position | proprioceptive capabilities, specifically position sense in the upper limb | *[7]* |

**Table S****2**. MRI acquisition parameters

|  | **T1w** | **dMRI** | **fMRI** |
| --- | --- | --- | --- |
| Sequence | 3D MPRAGE | spin-echo EPI sequence | 2D echo-planar imaging |
| Voxel (mm) | 0.8x0.8x0.8 | 1.5x1.5x1.5 | 2x2x2 |
| Matrix | 208x300x320 | 140x140 | 104x104 |
| Slices | 320 | 92 | 81 |
| TR (ms) | 2500 | 3230 | 800 |
| TE (ms) | 2.22 | 89.2 | 37 |
| TI (ms) | 1000 |  |  |
| Flip angle (deg) | 8 | 78 | 52 |
| Multiband factor | - | 4 | 8 |

**Table S****3**. Negative correlations between SC variable and SC canonical variate of the first significant mode (cognitive)

| **ROI 1** | **ROI 2** | **Correlation** |
| --- | --- | --- |
| Amygdala_L | Somatomotor_4L | -0.7 |
| Somatomotor_4R | DMN_temporal_2R | -0.67 |
| Somatomotor_3R | DMN_temporal_1R | -0.66 |
| Somatomotor_3R | Limbic_temporalPole_1R | -0.65 |
| VN_8L | FPN_precuneus_1L | -0.64 |
| Somatomotor_3R | DMN_temporal_2R | -0.63 |
| VAN_PFC_1L | DMN_PFC_5L | -0.63 |
| DAN_posterior_3L | FPN_precuneus_1L | -0.63 |
| Somatomotor_8R | DMN_ventral PFC_2R | -0.62 |
| VN_4L | Somatomotor_4L | -0.62 |
| VAN_medial_2R | DMN_ventral PFC_2R | -0.61 |
| DMN_precuneus PCC_2L | DMN_precuneus PCC_1R | -0.61 |
| Hippocampus_R | DMN_precuneus PCC_1L | -0.60 |
| VN_1L | Somatomotor_1R | -0.60 |
| Somatomotor_3R | FPN_parietal_2R | -0.59 |

Top 15 most negatively correlated brain connections shown only. DMN: default mode network. VN: visual network. FPN: frontoparietal network. PFC: prefrontal cortex. PCC: posterior cingulate cortex.

**Table S****4**. Positive correlations between SC variable and SC canonical variate of the first significant mode (cognitive)

| **ROI 1** | **ROI 2** | **Correlation** |
| --- | --- | --- |
| Limbic_OFC_1R | DMN_temporal_3R | 0.67 |
| VAN_medial_1L | FPN_parietal_1R | 0.66 |
| DMN_PFC_4L | DMN_parietal_1R | 0.66 |
| VAN_PFC_1L | Somatomotor_2R | 0.65 |
| Accumbens_L | VN_1R | 0.65 |
| VAN_PFC_1L | DMN_parietal_1R | 0.64 |
| Accumbens_L | VN_3R | 0.63 |
| Accumbens_L | DAN_posterior_1R | 0.63 |
| DAN_posterior_6L | VAN_medial_1R | 0.62 |
| Thalamus_L | DMN_parietal_1R | 0.62 |
| Somatomotor_2R | DMN_dorsal and medial PFC_1R | 0.61 |
| DMN_temporal_3R | DMN_dorsal and medial PFC_1R | 0.61 |
| Somatomotor_2R | FPN_parietal_1R | 0.6 |
| Accumbens_L | DMN_ventral PFC_2R | 0.6 |
| Accumbens_L | VN_4R | 0.6 |

Top 15 most positively correlated brain connections shown only. OFC: orbital frontal cortex. DMN: default mode network. VAN: ventral attention network. VN: visual network. FPN: frontoparietal network. PFC: prefrontal cortex. DAN: dorsal attention network.

**Table S****5**. Positive correlations between SC variables and SC canonical variate of the second significant mode (sensorimotor)

| **ROI 1** | **ROI 2** | **Correlation** |
| --- | --- | --- |
| Amygdala_L | VAN_temporalOccipitoparietal_1R | 0.64 |
| DAN_posterior_4R | DMN_ventral PFC_1R | 0.63 |
| DAN_posterior_3L | DMN_PFC_6L | 0.62 |
| DAN_posterior_3L | DMN_PFC_4L | 0.61 |
| DMN_temporal_1L | VAN_ temporalOccipitoparietal_1R | 0.6 |
| Somatomotor_3L | DAN_posterior_3L | 0.59 |
| DAN_posterior_5L | VAN_ temporalOccipitoparietal_1R | 0.59 |
| DAN_posterior_2R | DAN_posterior_5R | 0.58 |
| FPN_parietal_2R | FPN_lateral PFC_1R | 0.58 |
| Hippocampus_L | VAN_ temporalOccipitoparietal_1R | 0.58 |
| Somatomotor_3R | DAN_posterior_4R | 0.57 |
| Amygdala_L | DAN_posterior_3L | 0.56 |
| DAN_posterior_3L | DAN_precentral ventral_1L | 0.56 |
| DAN_posterior_5R | VAN_ temporalOccipitoparietal_1R | 0.56 |
| DAN_posterior_5R | DAN_precentral ventral_1R | 0.56 |

**Top 15 most positively correlated brain connections shown only.** DMN: default mode network. VAN: ventral attention network. FPN: frontoparietal network. PFC: prefrontal cortex. DAN: dorsal attention network.

**Table S****6**. Negative correlations between SC variables and SC canonical variate of the second significant mode (sensorimotor)

| **ROI 1** | **ROI 2** | **Correlation** |
| --- | --- | --- |
| DMN_parietal_1L | VAN_medial_2R | -0.54 |
| Cerebellum_L | Limbic_temporalPole_1L | -0.52 |
| VN_3R | Somatomotor_1R | -0.51 |
| DAN_FEF_1R | DMN_dorsal and medial PFC_3R | -0.5 |
| DMN_parietal_1L | DMN_PFC_7L | -0.49 |
| Cerebellum_R | Limbic_temporalPole_1L | -0.48 |
| Pallidum_L | Hippocampus_L | -0.47 |
| VAN_medial_1L | DMN_dorsal and medial PFC_1R | -0.46 |
| VAN_medial_3L | FPN_Cing_1L | -0.46 |
| DMN_parietal_1L | DAN_precentral ventral_1R | -0.46 |
| Accumbens_R | Limbic_temporalPole_2L | -0.46 |
| DMN_parietal_1L | DAN_FEF_1R | -0.45 |
| VN_3L | Limbic_temporalPole_2L | -0.45 |
| DMN_PFC_4L | FPN_lateral PFC_3R | -0.45 |
| VAN_medial_1L | FPN_lateral PFC_1R | -0.44 |

Top 15 most negatively correlated brain connections shown only. DMN: default mode network. VAN: ventral attention network. VN: visual network. FEF: frontal eye fields. FPN: frontoparietal network. PFC: prefrontal cortex. DAN: dorsal attention network.

**Table S****7**. Negative correlations between FC variables and FC canonical variate of the significant mode (sensorimotor)

| **ROI 1** | **ROI 2** | **Correlation** |
| --- | --- | --- |
| VN_1L | Limbic_temporalPole_1L | -0.57 |
| VN_1L | VN_1R | -0.55 |
| Hippocampus_L | DMN_precuneus PCC_1R | -0.54 |
| VN_6R | FPN_precuneus_1R | -0.54 |
| VN_4R | Somatomotor_5R | -0.53 |
| Somatomotor_5L | VN_4R | -0.53 |
| Hippocampus_L | DMN_precuneus PCC_1L | -0.53 |
| Somatomotor_5R | FPN_precuneus_1R | -0.53 |
| VN_4R | Somatomotor_6R | -0.52 |
| VN_1L | DMN_precuneus PCC_1R | -0.52 |
| Hippocampus_L | DMN_precuneus PCC_2R | -0.52 |
| Somatomotor_4L | FPN_precuneus_1R | -0.51 |
| VN_9L | FPN_precuneus_1R | -0.51 |
| Somatomotor_4L | DMN_precuneus PCC_2R | -0.5 |
| Somatomotor_4R | DMN_precuneus PCC_2R | -0.5 |

Top 15 most negatively correlated brain connections shown only. VN: visual network. DMN: default mode network. FPN: frontoparietal network. PCC: posterior cingulate cortex.

**Table S****8**. Positive correlations between FC variables and FC canonical variate of the significant mode (sensorimotor)

| **ROI 1** | **ROI 2** | **Correlation** |
| --- | --- | --- |
| DMN_PFC_3L | DMN_precuneus PCC_1R | 0.55 |
| DAN_precentral ventral_1R | FPN_parietal_1R | 0.53 |
| VAN_frontal operculum insula_1L | VN_8R | 0.53 |
| Amygdala_R | Accumbens_R | 0.53 |
| DAN_posterior_3R | DAN_precentral ventral_1R | 0.53 |
| VAN_frontal operculum insula_2L | DAN_posterior_3R | 0.52 |
| Accumbens_L | DAN_posterior_4L | 0.52 |
| VAN_frontal operculum insula_2L | VN_8R | 0.52 |
| Caudate_R | FPN_parietal_1R | 0.51 |
| VAN_parietalrOper_1L | VN_8R | 0.51 |
| FPN_parietal2R | FPN_medial posterior PFC_1R | 0.51 |
| VAN_frontal operculum insula_2L | VAN_temporalOccipitoparietal_2R | 0.49 |
| Caudate_R | Limbic_OFC_1R | 0.49 |
| DAN_precentral ventral_1L | VAN_frontal operculum insula_2L | 0.49 |
| Accumbens_L | Limbic_OFC_1R | 0.49 |

Top 15 most positively correlated brain connections shown only. DMN: default mode network. PFC: prefrontal cortex. PCC: posterior cingulate cortex. DAN: dorsal attention network. FPN: frontoparietal network. OFC: orbital frontal cortex. VAN: ventral attention network. VN: visual network.


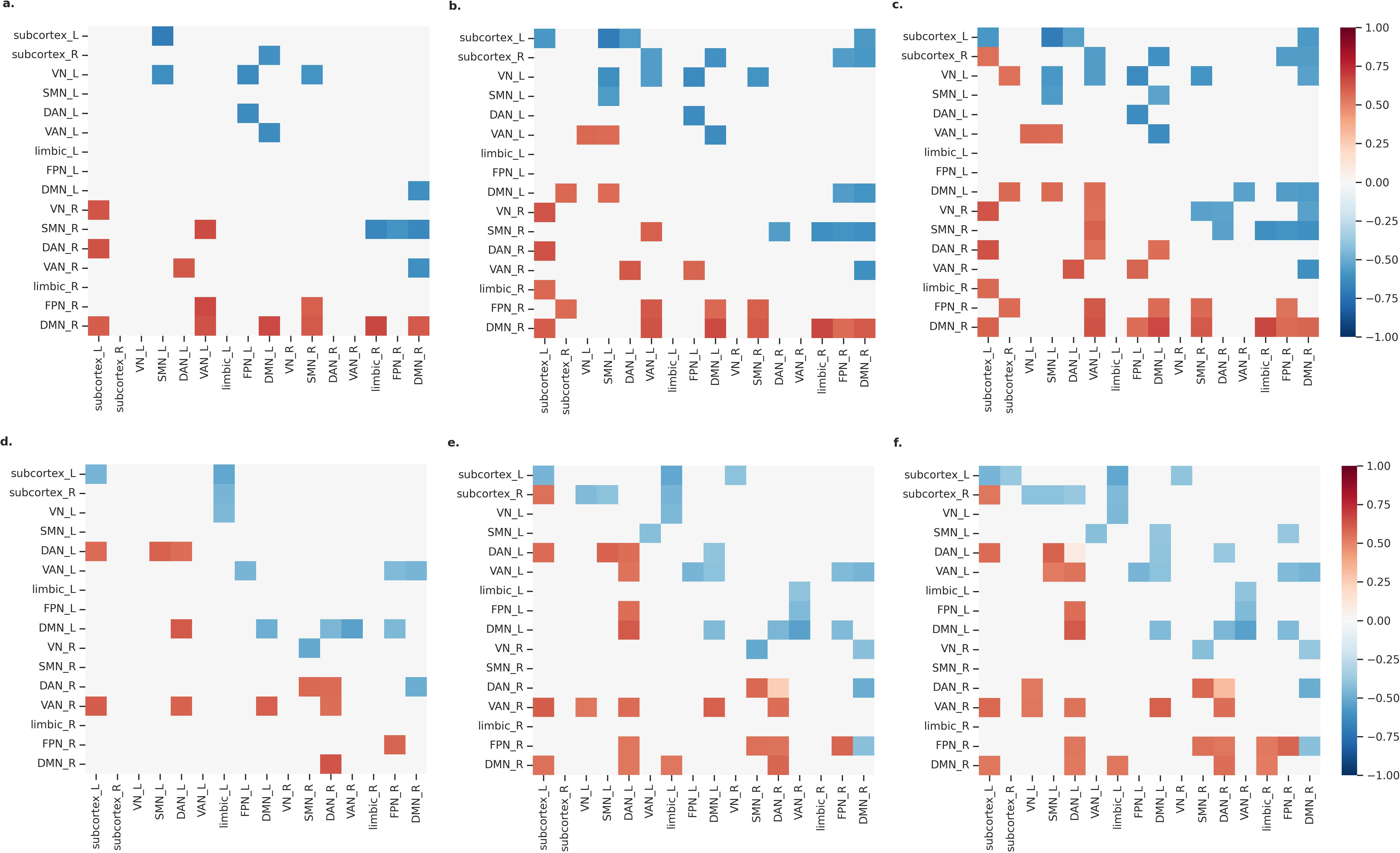
**Figure S****1**. Mean correlations between structural networks for the first (a-c) and the second (d-f) CCA mode at three different levels of top connections: top 15 (a, d), top 30 (b, e) and top 45 (c, f) of most positively/negatively correlated connections. Positive correlations (red) and negative correlations (blue) are summarized separately in the lower and upper triangular matrices, respectively. VN: visual network. SMN: somatomotor. DAN: dorsal attention network. VAN: ventral attention network. FPN frontoparietal network. DMN: default mode network.


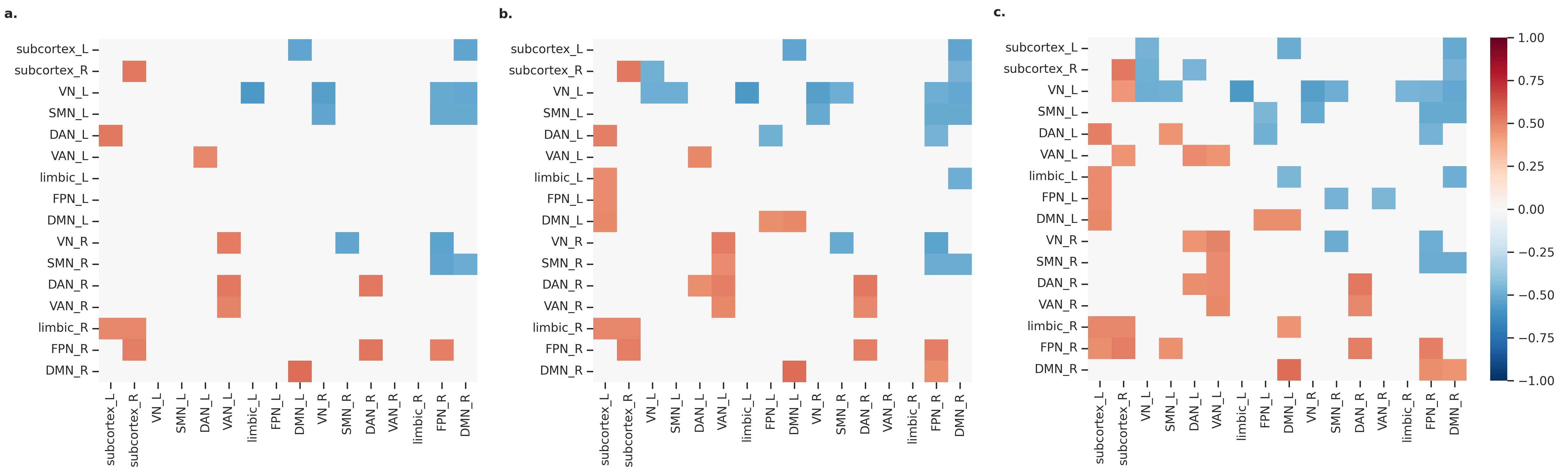
**Figure S****2**. Mean correlations between functional networks for the CCA mode at three different levels of top connections: top 15 (a), top 30 (b) and top 45 (c) of most positively/negatively correlated connections. Positive correlations (red) and negative correlations (blue) are summarized separately in the lower and upper triangular matrices, respectively. VN: visual network. SMN: somatomotor. DAN: dorsal attention network. VAN: ventral attention network. FPN frontoparietal network. DMN: default mode network.

**Table S****9**. Multiple regression model structural connectivity

|  | **First SC-Kinarm mode** | | |  | Second SC-Kinarm mode | | |
| --- | --- | --- | --- | --- | --- | --- | --- |
|  | **Canonical variate** | **Duration of epilepsy** | **Education level** |  | **Canonical variate** | **Duration of epilepsy** | **Education level** |
| **Coefficient** | 0.65 | -0.0085 | -0.0029 |  | 0.2 | 0.01 | 0.0036 |
| **Std error** | 0.13 | 0.015 | 0.071 |  | 0.08 | 0.008 | 0.035 |
| **t-statistic** | 5 | -0.56 | -0.04 |  | 2.5 | 1.3 | 0.101 |
| **‍p value** | **< 0.001** | 0.59 | 0.97 |  | **0.04** | 0.19 | 0.92 |

**Table S****10**. Multiple regression model functional connectivity

|  | **FC-Kinarm mode** | | |
| --- | --- | --- | --- |
|  | **Canonical variate** | **Duration of epilepsy** | **Education level** |
| **Coefficient** | 0.21 | -0.02 | -0.007 |
| **Std error** | 0.05 | 0.01 | 0.05 |
| **t-statistic** | 4.2 | -2 | -0.14 |
| **‍p value** | **0.002** | 0.06 | 0.9 |
